# Supplementary material for: A Randomised, Comparative, Effectiveness Trial Evaluating Low- versus High-Level Supervision of an Exercise Intervention for Women with Breast Cancer: The SAFE Trial
Source: Cancers (Basel). 2022 Mar 16;14(6):1528. doi: 10.3390/cancers14061528 (PMC8946819; doi:10.3390/cancers14061528)
Supplement: Supplementary file 1 [file cancers-14-01528-s001.zip › SAFE_Online Table S1_7Feb2022.pdf]

Online Table S1. Changes to study protocol following trial registration

TITLE: A comparative, effectiveness trial evaluating high- versus low-level supervision of an exercise intervention for women with breast cancer: the SAFE trial.

Rosalind R Spence [a], Carolina X Sandler [b, a, c], Benjamin Singh [d], Jodie Tanner [d], Christopher Pyke [e, f], Elizabeth Eakin [g], Dimitrios Vagenas [h], Sandra C Hayes [a]

a. Menzies Health Institute Queensland, Griffith University, Queensland, Australia.
b. School of Health Sciences, University of Western Sydney, New South Wales, Australia
c. Kirby Institute, UNSW Sydney, New South Wales, Australia.
d. Institute of Health and Biomedical Innovation, Queensland University of Technology, Queensland, Australia
e. Mater Health Services, South Brisbane, Queensland, Australia
f. Mater Clinic Unit Faculty of Medicine, The University of Queensland, Queensland, Australia.
g. School of Public Health, Faculty of Medicine, The University of Queensland, Queensland, Australia.
h. School of Public Health and Social Work, Faculty of Health, Queensland University of Technology, Queensland, Australia

Corresponding author: Rosalind Spence

Mailing address: G40 Griffith Health Centre, Level 8.86 Gold Coast campus Griffith University, QLD 4222

Telephone: +61 (0)7 3735 5194

E-mail: r.spence@griffith.edu.au

Online Resource Table S1: Changes to study protocol following trial registration

Original Protocol [1]	Modified Protocol [2]	Justification / Impact
Study design		
Modified wait-list control trial: the active treatment group will receive the intervention for 12 weeks following the baseline assessment. During this period the wait-list control group will receive no intervention. At 12 weeks both groups will complete an assessment of primary and secondary outcomes. Following this the active treatment group will receive no further intervention, while the wait-list control group receive a modified intervention (i.e., the exercise volume will be identical to the original intervention; however, it will be delivered via a less supervised model of care). Both groups will complete the same assessments 12 weeks later (i.e., 24 weeks after the baseline assessment).	We removed the wait-list component.<br><br>The participants were randomised into one of two exercise intervention groups immediately following baseline assessment. Both groups received support from an exercise professional to reach the exercise target dosage of 150 minutes of moderate-intensity exercise per week. The groups differed only according to the number of supervised sessions they were offered across the 12-week intervention. Participants in the High-supervision group were allocated 20 sessions (original active treatment group), while the Low-supervision group were allocated five face-to-face sessions (original modified intervention which was to be delivered weeks 12-24), over the 12-week intervention period (weeks 1-12).<br><br>Both groups received no further intervention between weeks 12-24.	n=35 women declined to participate in the study and study staff identified the delay to commencing exercise if randomised to the wait-list (active) control group as a major deterrent to joining the program (see Consort flow chart). Given that the primary outcomes were safety and feasibility, the presence of a usual care control group was unnecessary. Transitioning the wait-list to a concurrent comparative group removed the requirement for delaying exercise commencement for the 5-session group but maintained the ability to explore safety and feasibility and compare efficacy.
Inclusion criteria		
Safety<br>The exercise intervention will be deemed as 'safe' when there is:<br>(i) no worsening of current treatment-related side effects,<br>(ii) no increase in medication(s) prescribed for chronic disease(s), and	Adverse events (safety outcome) were defined in accordance with the Good Clinical Practice Guidelines [15] as 'any unfavourable and unintended sign, symptom or disease that occurs in a participant whether it is considered to be study- or non-study-related'. Adverse events were deemed to be exercise-related if they occurred during or within two hours of	The pragmatic nature of this trial led to questioning of the validity of the data related to outcome i and ii. In contrast, the data collection protocol minimised any impact of recall bias related to outcome iii, and it was

(iii) absence of exercise-related adverse event whereby the participant could not participate in the intervention for two or more weeks.	supervised or unsupervised exercise or had a clear mechanism relating the adverse events to exercise (as determined by treating medical team or senior Exercise Professional). The *a priori* acceptable threshold for safety was set at no grade 3 or above exercise-related adverse events.	therefore deemed the most appropriate and valid outcome for trial safety.<br>All exercise-related adverse events were collected, regardless of impact on intervention.
All participants must be undergoing or have completed treatment within the past 24 months, with the exception of hormone therapy.	Currently undergoing treatment or completed treatment within the past **five** years	This criterion was unnecessarily restricting the size of the population from which we were sampling and was restricting our ability to recruit the needed sample size in a timely fashion. The key characteristics of the target population were stage of disease, baseline exercise levels and the presence of comorbidities and/or persistent treatment-related side effects. Women who met these criteria despite being 2-5 years post-treatment would be appropriate participants.
Study details		
Date of first participant enrolment<br>Actual<br>2/05/2016	Actual<br>2/06/2016	This was a typographical error in entering the date of the first participant.

[1] Protocol as per ANZCTR registration (ACTRN12616000547448), April 2016. [2] Protocol as implemented
