# Supplementary material for: A Randomised, Comparative, Effectiveness Trial Evaluating Low- versus High-Level Supervision of an Exercise Intervention for Women with Breast Cancer: The SAFE Trial
Source: Cancers (Basel). 2022 Mar 16;14(6):1528. doi: 10.3390/cancers14061528 (PMC8946819; doi:10.3390/cancers14061528)
Supplement: Supplementary file 1 [file cancers-14-01528-s001.zip › SAFE_Online Table S2_7Feb2022.pdf]

Online Table S2. Key Differences Between Exercise Intervention Groups

TITLE: A comparative, effectiveness trial evaluating high- versus low-level supervision of an exercise intervention for women with breast cancer: the SAFE trial.

Rosalind R Spence [a], Carolina X Sandler [b, a, c], Benjamin Singh [d], Jodie Tanner [d], Christopher Pyke [e, f], Elizabeth Eakin [g], Dimitrios Vagenas [h], Sandra C Hayes [a]

a. Menzies Health Institute Queensland, Griffith University, Queensland, Australia.
b. School of Health Sciences, University of Western Sydney, New South Wales, Australia
c. Kirby Institute, UNSW Sydney, New South Wales, Australia.
d. Institute of Health and Biomedical Innovation, Queensland University of Technology, Queensland, Australia
e. Mater Health Services, South Brisbane, Queensland, Australia
f. Mater Clinic Unit Faculty of Medicine, The University of Queensland, Queensland, Australia.
g. School of Public Health, Faculty of Medicine, The University of Queensland, Queensland, Australia.
h. School of Public Health and Social Work, Faculty of Health, Queensland University of Technology, Queensland, Australia

Corresponding author: Rosalind Spence

Mailing address: G40 Griffith Health Centre, Level 8.86 Gold Coast campus Griffith University, QLD 4222

Telephone: +61 (0)7 3735 5194

E-mail: r.spence@griffith.edu.au

Online Resource Table S2. Key Differences Between Exercise Intervention Groups

Exercise Intervention Characteristic	Exercise Intervention Groups	
	**Low-supervision group**	**High supervision group**
Length of intervention, weeks	12	12
Total number of supervised sessions during intervention	5	20
Frequency of supervised sessions per week		
Week 1	1	2
Week 2 – 8	Determined by ExP and Pt	2
Week 9 – 12	Determined by ExP and Pt	1

ExP: Exercise professional; Pt: participant
