# Supplementary material for: A Randomised, Comparative, Effectiveness Trial Evaluating Low- versus High-Level Supervision of an Exercise Intervention for Women with Breast Cancer: The SAFE Trial
Source: Cancers (Basel). 2022 Mar 16;14(6):1528. doi: 10.3390/cancers14061528 (PMC8946819; doi:10.3390/cancers14061528)
Supplement: Supplementary file 1 [file cancers-14-01528-s001.zip › SAFE_Online Table S3_7Feb2022.pdf]

Online Table S3. Principles of exercise prescription and reporting used in the SAFE exercise intervention (based on Consensus on Exercise Reporting Template)

TITLE: A comparative, effectiveness trial evaluating high- versus low-level supervision of an exercise intervention for women with breast cancer: the SAFE trial.

Rosalind R Spence [a], Carolina X Sandler [b, a, c], Benjamin Singh [d], Jodie Tanner [d], Christopher Pyke [e, f], Elizabeth Eakin [g], Dimitrios Vagenas [h], Sandra C Hayes [a]

a. Menzies Health Institute Queensland, Griffith University, Queensland, Australia.
b. School of Health Sciences, University of Western Sydney, New South Wales, Australia
c. Kirby Institute, UNSW Sydney, New South Wales, Australia.
d. Institute of Health and Biomedical Innovation, Queensland University of Technology, Queensland, Australia
e. Mater Health Services, South Brisbane, Queensland, Australia
f. Mater Clinic Unit Faculty of Medicine, The University of Queensland, Queensland, Australia.
g. School of Public Health, Faculty of Medicine, The University of Queensland, Queensland, Australia.
h. School of Public Health and Social Work, Faculty of Health, Queensland University of Technology, Queensland, Australia

Corresponding author: Rosalind Spence

Mailing address: G40 Griffith Health Centre, Level 8.86 Gold Coast campus Griffith University, QLD 4222

Telephone: +61 (0)7 3735 5194

E-mail: r.spence@griffith.edu.au

Online Resource Table S3. Principles of exercise prescription and reporting used in the SAFE exercise intervention (based on Consensus on Exercise Reporting Template (13[a]))

**Principles**	
**Application in SAFE**	**Notes**
**Exercise prescription**	
**Dosage**	
Intervention target: 150 minutes per week of exercise at moderate or above intensity<br>= 150 minutes x 4 METs = 600 metabolic equivalent of task minutes (MET-mins) per week.	Weekly exercise dosage was the prescription goal of the intervention. It was expected that some participants would need to gradually progress towards this target (progression could take 1-12+ weeks) and for some participants this target may never be achievable. Individual weekly dosage was tailored to the individual by the ExP.
**Frequency / Duration**	
150 minutes, of at least moderate-intensity exercise (aerobic and resistance), or equivalent duration of higher-intensity exercise (weekly volume target = 600 MET-mins)<br><br>Frequency of sessions ranged from 3-7 sessions/week, with a target of at least 2 resistance sessions per week.	Individual exercise sessions ranged from 5 minutes to 90 minutes.<br>Resistance sessions had 2-8 exercises, 1-4 sets with 5-20 repetitions per set.
**Intensity**	
Moderate intensity or higher (Rating of Perceived Exertion [RPE] 12-14+)	Exercise Professionals (ExP) taught participants to use the RPE scale in the first sessions, explaining the use of RPE in prescribing and monitoring exercise intensity, as well as reporting completed exercise. To assist participants in using the RPE scale, ExP anchored moderate intensity to the “talk test” (aerobic sessions) or repetitions in reserve (strength sessions).
**Description of exercise**	

In line with physical activity guidelines, no set exercises were specified. Participants were provided with a mixed-mode (aerobic and resistance) program, individually tailored to their exercise mode preferences, function and goals of the individual.<br><br>Aerobic exercise targeted cardiovascular function and function capacity.<br><br>Resistance exercise targeted major muscle groups and any rehabilitation of specific concern for an individual (e.g., knee pain)	An individualized, printed or electronic, exercise program was created for each individual and progressed as needed.<br><br>No specific exercise type was excluded from potential inclusion; however, the ExP would advise for or against specific exercises based on clinical judgement of safety, specificity for achieving goals and participant mode of preference to maintain engagement. It was expected that walking would be the primary type of aerobic exercise and body-weight or exercise-band (e.g., Theraband®) based exercises would provide the foundation of the resistance sessions for the majority of participants.<br><br>During periods of intense symptoms (e.g., fatigue or nausea), prescriptions included sessions broken up into multiple bouts, including repetitive seated movements similar to low-load / high-repetition resistance exercises (e.g., shoulder press without weights).
**Tailoring**	
Although the intervention target was generic, it was prescribed to each participant in an individualised manner, taking into consideration individual goals, health issues (side-effects, comorbidities) and response to exercise. Modifications could apply to the weekly volume (e.g., intensity or duration may not reach target in any given week) and inclusion of aerobic and resistance exercise (i.e., some participants did not complete either aerobic or resistance exercise for various reasons).<br><br>Furthermore, the target intervention was intentionally broad in order to reflect the current guidelines. The type of exercise and the frequency, intensity and duration of exercise that was used to reach the prescribed volume was individualised for each participant.	Prescribing exercise that was appropriate, safe and focussed on any given participant's goals was the primary intention. Where appropriate, ExP encouraged and supported the participant to reach the intervention weekly target dosage, through specific exercises that suited the participant's function and preferences.<br><br>How the content was covered, and the content of any given session was determined by the ExP and influenced by the needs of the participant and the frequency of the sessions.

**Starting level**	
Starting exercise level (e.g., weekly volume, session intensity and duration, mode) was participant dependent, based on clinical judgement of prescribing ExP, and considering recent exercise history and symptom response.<br><br>Note: All participants were completing <150 minutes of structured exercise at enrolment into the study.	Participants were not expected to reach the target dosage in week 1 (unless appropriate) and individual weekly prescription progressed towards this goal based on the clinical judgement of the ExP (and reporting of symptom response to previous week exercise prescription).
**Exercise Progression**	
Prescription adhered to the exercise principles of gradual progression as participants progressed from baseline exercise volumes to the target dosage.<br><br>Exercise was to be completed at a perceived intensity of at least moderate (RPE 11-13). If a participant's symptom-burden reduced or fitness increased, exercise prescription was progressed to maintain the target intensity of the exercise. Inversely, if symptoms increased or capacity decreased then exercise prescription was regressed to maintain intensity based on perceived exertion.<br><br>Progression/ regression of exercise was based on clinical judgement of prescribing ExP, considering recent exercise history and symptom response.	Progression/ regression involved modifying speed, load, duration, number of exercises, rest breaks, and/ or number of bouts.
**Overload**	
Protocol was to progress each participant from baseline exercise levels (<150 minutes) to at least 150 minutes of moderate+ intensity, mixed mode unless there were clinical reasons for not reaching the target. For participants who were meeting the target dosage (equivalent to 600 MET-mins / week), it was expected that exercise prescription would continue to include overload (e.g., increased duration, speed or load) to maintain an RPE of moderate-intensity. If clinically appropriate,	Maintaining an RPE of moderate intensity in the presence of physical adaptations to exercise requires an increase in exercise dose (e.g., increased exercise speed, load, session duration and/ or weekly duration). However, in this population, the absolute dose of exercise may not have appeared to be increasing because of the impact of physical decline and/or presence of symptoms. For example, during periods of high symptom burden, RPE may have increased without any concomitant increase

participants could be prescribed greater than 600 MET-mins / week.	in exercise dose. ExPs are trained to respond to reduced RPE due to adaptations and increased RPE due to symptoms and / or deconditioning with prescription progression and regression, respectively.
**Specificity**	
Mixed aerobic and resistance exercise was prescribed, as recommended by the exercise and cancer guidelines. This allowed for the evaluation of the safety and feasibility of the guidelines in this population.	ExP tailored the prescription to needs or goals specific to the individual (including comorbidities and side-effects) within the parameters of the intervention target.
**Initial value**	
Eligibility restricted sample to only those completing < 150 minutes of weekly exercise (self-report) at recruitment.	Primary outcomes were safety and feasibility; therefore, it was important to target a population who were not currently completing the intervention prescription.
**Reversibility / Diminishing returns**	
Follow-up assessment 12 weeks post-intervention completion	
**Intervention details (WHO, WHERE, HOW)**	
**Type of exercise equipment**	
Participant preference, including:<br>Aerobic: walking, aerobic-style videos or classes or circuits, ergometers (treadmills, stationary bike etc)<br>Resistance: Body weight, free weights, gym-based machine weights, graded exercise bands (e.g., Theraband®)	No type of equipment was excluded from use. ExP made suggestions based on each participant's goals, access to equipment and contraindications
**Qualifications of exercise instructor**	
Exercise Professionals were all Accredited Exercise Physiologists with study-specific training and supervision by senior ExPs with >10 years of exercise-oncology experience.	"Accredited Exercise Physiologist" is an Australian qualification. They are registered allied health professionals who have completed a tertiary-level 4-year bachelor's degree or equivalent. Core curriculum includes oncology-specific education.
**Group / individual**	
Individual	Participants were not restricted from taking part in group-based training (e.g.,

	community- or clinic-based group sessions) outside of the supervised sessions provided as part of this study.
**Supervision**	
Face-to-face delivery. The protocol was not prescriptive, but the supervised sessions generally included a resistance component and an educational / motivational component.<br><br>High-supervision: 20 supervised sessions with an ExP (2/week for weeks 1-8;, 1/week for weeks 9-12)<br><br>Low-supervision: 5 supervised sessions with an ExP (1 session in week 1, subsequent sessions scheduled by ExP throughout weeks 2-12). Supervision also included ensuring correct exercise technique, monitoring exercise intensity, discussing progress, and adjusting the exercise program as necessary.	Participants could have independently-sourced and/or privately-funded supervised sessions in addition to study sessions.
**Adherence/ Compliance recording**	
Participants were asked to record all exercise completed on a daily basis in a provided, structured exercise-tracker booklet. Data was checked and recorded by ExP during each supervised session.	Data on each session included: mode of exercise, intensity of exercise (RPE scale, 6-20), duration of exercise, number of bouts session was broken into (if applicable).
**Motivational strategies and non-exercise components**	
Behaviour change strategies (exercise counselling) underpinned exercise intervention. Sessions and prescription were based on the Chronic Disease Self-Management model (modified “4 A’s”), ensuring participant needs were identified and met (14).	Education and self-management were integral components, including benefits of exercise to participant’s situation and how to start and progress exercise (with the goal of supporting independent exercise). Goal-setting and overcoming barriers and problem-solving were components of all sessions.
**Home program component**	
The home program was prescribed and instructed during supervised sessions and included aerobic and resistance components to be completed across multiple	Additional balance, stretching and pelvic floor exercises were prescribed for the home program as appropriate (not counted in total duration).

sessions/bouts/days as appropriate for the individual.	
**Type and number of AE**	
Comprehensively collected via regular self-report.<br><br>[Primary outcome: See results for safety outcomes]	Adverse events, both treatment- / disease-related and exercise-related were reported utilising the Common Terminology Criteria for Adverse Events (CTCAE), version 4 grading system and standardized language (Medical Dictionary for Regulatory Activities [medDRA]).
**Location**	
A convenient location chosen by participant.	e.g., home, gym, park
**Fidelity**	
Implementation of the exercise intervention protocol was monitored throughout the study by senior staff, via auditing of case management notes, attendance at random sessions and case discussions.<br><br>[Primary outcome: see results for compliance to the exercise target (exercise volume)]	Compliance was defined as weekly volume of exercise completed and completion of minimum number of resistance sessions per week.

ExP – exercise professional; RPE – rate of perceived exertion; MET-mins - metabolic equivalent of task minutes (a measure of exercise volume calculated as duration x intensity).

[a] Reference 13: Slade SC, Dionne CE, Underwood M, Buchbinder R (2016) Consensus on Exercise Reporting Template (CERT): Explanation and Elaboration Statement. Br J Sports Med 50 (23):1428-1437. doi:10.1136/bjsports-2016-096651
