# Supplementary material for: A Randomised, Comparative, Effectiveness Trial Evaluating Low- versus High-Level Supervision of an Exercise Intervention for Women with Breast Cancer: The SAFE Trial
Source: Cancers (Basel). 2022 Mar 16;14(6):1528. doi: 10.3390/cancers14061528 (PMC8946819; doi:10.3390/cancers14061528)
Supplement: Supplementary file 1 [file cancers-14-01528-s001.zip › SAFE_Online Table S4_7Feb2022.pdf]

## Online Table S4: Frequency of grade 1-2 exercise-related adverse events

**TITLE:** A comparative, effectiveness trial evaluating high- versus low-level supervision of an exercise intervention for women with breast cancer: the SAFE trial.


Rosalind R Spence [a], Carolina X Sandler [b, a, c], Benjamin Singh [d], Jodie Tanner [d], Christopher Pyke [e, f], Elizabeth Eakin [g], Dimitrios Vagenas [h], Sandra C Hayes [a]

a. Menzies Health Institute Queensland, Griffith University, Queensland, Australia.
b. School of Health Sciences, University of Western Sydney, New South Wales, Australia
c. Kirby Institute, UNSW Sydney, New South Wales, Australia.
d. Institute of Health and Biomedical Innovation, Queensland University of Technology, Queensland, Australia
e. Mater Health Services, South Brisbane, Queensland, Australia
f. Mater Clinic Unit Faculty of Medicine, The University of Queensland, Queensland, Australia.
g. School of Public Health, Faculty of Medicine, The University of Queensland, Queensland, Australia.
h. School of Public Health and Social Work, Faculty of Health, Queensland University of Technology, Queensland, Australia

**Corresponding author**: Rosalind Spence

Mailing address: G40 Griffith Health Centre, Level 8.86 Gold Coast campus Griffith University, QLD 4222

Telephone: +61 (0)7 3735 5194

E-mail: r.spence@griffith.edu.au

Online Resource Table S4: Frequency of grade 1-2[a] exercise-related adverse events

| Category / Description* | Total (n=126) | High (n=103) | Low (n=23) |
|---|---|---|---|
| **Musculoskeletal and connective tissue disorders** | | | |
| Myalgia (including delayed onset muscle soreness) | 56 | 37 | 11 |
| -Back pain | 8 | 4 | 4 |
| Muscle cramp | 2 | 2 | - |
| Arthralgia | 1 | 1 | - |
| Joint effusion | 1 | 1 | - |
| **General Disorders** | | | |
| Pain | 40 | 33 | 7 |
| Fatigue | 9 | 8 | 1 |
| Edema | 2 | - | 2 |
| Lymphoedema | 2 | 2 | - |
| Flu-like symptoms | 2 | 2 | - |
| **Nervous system disorders** | | | |
| Headache | 3 | 3 | - |
| Dizziness | 5 | 3 | 2 |
| Presyncope | 1 | 1 | - |
| **Respiratory disorders** | | | |
| Bronchospasm | 1 | 1 | - |
| Cough | 1 | 1 | - |
| **Injury** | | | |
| Bruising | 1 | 1 | - |
| Fall | 1 | 1 | - |
| **Gastrointestinal disorders** | | | |
| Gastroesophageal reflux disease | 1 | 1 | - |
| Nausea | 2 | 2 | - |
| **Skin and subcutaneous tissue disorders** | | | |
| Nail changes | 1 | 1 | - |
| Skin infection | 1 | 1 | - |
| **Other (non-CTCAE terminology)** | | | |
| Cording | 1 | 1 | - |
| **TOTAL** | **126** | **103** | **23** |

CTCAE: Common Terminology Criteria for Adverse Events

[a] No grade three or above adverse events occurred during the trial
